# Tertiary Survey in the Days of Modern Imaging: Assessing the Detection Rate of Clinically Significant Injuries on Tertiary Survey in a Level 2 Trauma Centre

**DOI:** 10.7759/cureus.21962

**Published:** 2022-02-06

**Authors:** Kirk H Underwood, Emily Doole, Daniel Breen, Glenn Guest, David Watters, Eileen M Moore, Sonal Nagra

**Affiliations:** 1 Department of Surgery, Barwon Health, Geelong, AUS

**Keywords:** selective ct, trauma ct, tertiary survey, whole body ct scan, pan scan

## Abstract

Aim: To determine the utility of tertiary survey (TS) in patients subjected to whole-body CT (WBCT) or selective CT (SCT) following trauma.

Methods: A retrospective analysis was performed on trauma patients admitted to a level 2 trauma centre following the introduction of a standardised TS form in 2017. The initial imaging protocol (WBCT versus selective CT versus x-ray), subsequently requested imaging, standardised injury data, and length of stay (LOS) were recorded. Clinically significant injuries were defined as those with an Injury Severity Score (ISS) of 1 on the Abbreviated Injury Scale (AIS).

Results: Five hundred and seven patients were included. The rate of additional significant injuries at the time of TS was 1.18% (n=6), each requiring conservative management only. There was no significant difference in injury detection based on the initial imaging protocol; however, there were three near-misses identified. Of these patients, two underwent selective CT and one was subjected to a plain film series, with clinically significant injuries identified early upon completion of trauma imaging. Overall, 2.9% (n=15) of patients had completed trauma imaging during the same admission. WBCT was associated with higher ISS and length of stay (p<0.05). After controlling for ISS, there was no difference in length of stay between imaging modalities except in those patients with an ISS of 0 (no clinically significant injuries), who appeared to have longer admissions if subject to WBCT (p<0.001).

Conclusion: The rate of missed injuries identified at TS is low. The imaging modality did not alter this. This may allow for the omission of the tertiary survey and earlier discharge in many trauma patients.

## Introduction

University Hospital Geelong (UHG) in Victoria, Australia, is a level 2 trauma centre servicing a large regional population of approximately 500,000 people. UHG sees approximately 13,000 traumas in the emergency department per year, with 25% of these admitted to hospitals [1,2]. A large proportion of these patients are admitted for observation and tertiary survey (TS) to assess non-life-threatening injuries that may have been missed earlier due to distracting injuries, intoxication, or busy environments.

Modern advances in radiology have greatly affected how trauma patients are assessed at presentation. The use of eFAST ultrasound and diagnostic adjuncts such as X-ray and CT scanning means that the majority of patients are subject to some form of imaging in the trauma setting.

Debate exists over the place of whole-body CT (WBCT; vertex to pubic symphysis) in the assessment of trauma patients. Proponents argue for a decrease in time-consuming and risky transfers, decreased time in the emergency department, and a good injury detection rate [3]. Ionising radiation remains a real concern in the application of this technology, with many arguing for a selective CT (SCT) approach. In SCT, clinical assessment combined with point-of-care ultrasound and trauma bay X-ray is employed to direct CT imaging to only the regions of interest, reducing radiation [4].

To date, studies assessing the effects of the imaging approach on the rates and magnitude of clinically significant injuries have been focused on level 1 centres and have included only major trauma [3]. The objective of this study was to determine the effect of an early imaging protocol on the rate of missed injuries. As a secondary outcome, we evaluated the effects of early imaging on length of stay (LOS) and the need for completion trauma imaging to further quantify patient radiation risk.

## Materials and methods

A retrospective analysis was performed on patients who underwent TS at University Hospital Geelong following the introduction of a standardised TS form. Clinical audit approval was granted by the Barwon Health Internal Review Board, having met State and Federal requirements for ethical research. Patient episodes were extracted from the hospital database based on form completion. This query extracted 1200 patient episodes between 2017 and 2019, with a significant amount of data lost due to a 2019 ransomware attack [5]. We believe that data loss did not systematically bias our results. 

Analysis was performed by a qualified statistician to ensure the study was adequately powered. Calculations showed that 395 patients were required for analysis assuming a conservative expected proportion of 0.5% missed injuries on TS [6] with a 95% confidence interval and a 5% margin of error.

TS was performed by registrars and senior resident medical officers with general registration. The standardised TS included a checklist of areas for review to ensure a reproducible approach to the TS, including a complete review of available radiology. The data extracted through this retrospective analysis included mechanisms, injuries detected at initial assessment (primary and secondary surveys), injuries detected on TS, timing of TS, length of stay, injury severity as per Abbreviated Injury Scale (AIS), and imaging approach. Where available, further data on missed injuries leading to representation or detected after a tertiary survey was collected, however, this information was not systematically documented.

The imaging approach was categorised into WBCT, SCT, ‘plain film’ or ‘no imaging’. Admission episodes were further investigated to determine if additional advanced imaging was performed, with a specific focus on whether completion trauma imaging was needed for clinical decision making.

Statistical analysis was performed to determine the rate of clinically significant injuries (missed injuries) detected on TS, defined as those scoring an Injury Severity Score (ISS) of >1 on the AIS. Lacerations repaired in the theatre were recorded, however, those repaired in the emergency department, where documentation is not standardised, were excluded.

## Results

Missed injuries

**Figure 1:**
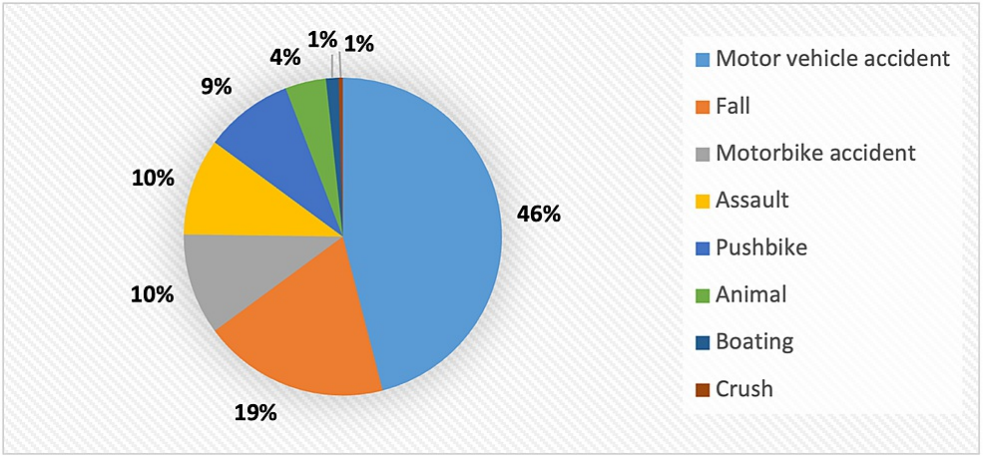
Trauma mechanisms.

**Figure 2:**
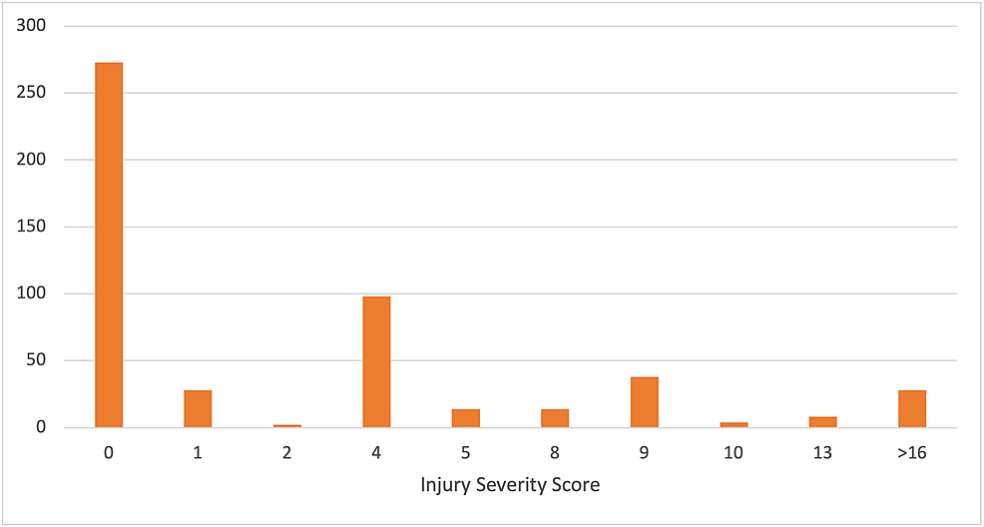
Injury Severity Score by frequency of occurrence.

A detailed analysis was performed on 507 trauma patients at UHG. 38% (n= 192) of these traumas were female. The mean age of the patients was 41 years old. The majority of admissions were related to road trauma, with 284 motorised collisions identified (Figure 1). The ISS ranged from 0 to 34, with a mean of 3.3. The majority (n=273) of trauma patients who underwent TS had no clinically significant injuries, with an ISS of 0 (Figure 2). There were no deaths within 30 days for this group.

A total of six (1.18%) clinically significant missed injuries were identified on TS. These injuries, outlined in Table 1, included three dental fractures, a grade 1 acromioclavicular joint dislocation, a humeral condylar fracture, and an undisplaced pubic rami fracture. Dental injuries were not subsequently managed at UHG, and both fractures were managed conservatively. Only the undisplaced pubic rami fracture was within the scope of the WBCT protocol, and it was confirmed on radiological review of the initial CT following clinical assessment.

**Table 1:** Types of injuries identified on tertiary survey. WBCT: whole body CT, MVA: motor vehicle accident, MBA: motor bike accident.

Injury identified on tertiary	Abbreviated Injury Score of missed injury	Total ISS before tertiary	Total ISS after tertiary	Imaging	Mechanism
Dental fracture	1	1	1	WBCT	Animal
Comminuted distal humeral lateral condylar fracture	3	29	29	WBCT	Assault
Dental fracture	1	9	9	WBCT	MBA
Dental fracture	1			WBCT	Boating
Undisplaced pubic rami fracture	2	4	8	WBCT	MVA
Grade 1 AC joint dislocation	2	4	4	WBCT	Mountain Bike

Four additional clinically significant injuries were identified during the course of this study, which led to representation at University Hospital (Table 2). These injuries were either identified due to representation with non-resolving pain, or after internal review of imaging post discharge. This number does not take into account injuries identified in the primary care setting or elsewhere.

**Table 2:** Missed injuries not identified on tertiary survey, but identified upon representation to hospital. MVA: motor vehicle accident, SCT: selective CT.

Missed injury identified	Abbreviated Injury Score	ISS before missed injury	ISS after missed injury	Imaging	Mechanism	Cause of missed injury
Spinal injury misreported as stable and on review of imaging deemed unstable.	3	24	29	WBCT	MVA	Formal radiology misreported and identified on internal review
Left sacral ala fracture	2	13	13	WBCT	Car versus pedestrian	Fracture not apparent on CT. Re-presented with pain and detected on MRI.
AC joint dislocation	2	0	4	SCT (C spine) and plain films	MVA	Discharged before radiologist review, patient recalled for treatment.
Second metatarsal head fracture	2	0	4	Plain films (including foot)	Fall from roof	Fracture not apparent on plain film.

Effects of imaging

Additional Investigations

All patients who had missed injuries identified on TS were assessed with a WBCT scan as the initial imaging modality, with no missed injuries due to SCT, plain film, or clinical assessment alone groups (Table 1). Of the patients that re-presented with missed injuries (i.e., negative TS), all had appropriately targeted imaging of the injury site. The error was either due to misreporting, discharge before consultant radiologist review (in the case of plain films), or lack of imaging sensitivity (Table 2).

A total of 492 patients had some form of imaging as an adjunct to the secondary survey. eFast ultrasound results were not reliably recorded and were excluded from this study. Approximately 9.7% (n=48/492) of patients had additional CT imaging either for completion of trauma assessment (3%, n=15/492) or subspecialty planning (6.7%, n=33, Table 3).

**Table 3:** Additional CT scans performed during index admission grouped by initial imaging modality.

	First imaging modality				
Next additional imaging	WBCT	Selective	Plain films alone	No imaging	Grand total
No additional CT	225	170	49	15	459
Completion trauma CT		9	6		15
Orthopaedic CT	14	4	2		20
Serial trauma CT	8	1			9
Vascular CT		2			2
Urology (CTIVP)		2			2
Total	247	188	57	15	507

Three ‘near misses’ were identified during the course of this study, with injuries detected early and prior to the tertiary survey due to completion trauma imaging (Table 4). Two of these were associated with SCT as the initial imaging modality, and one was initially assessed with plain films, the latter being a paediatric patient.

**Table 4:** Additional injuries detected following early initiation of additional CT imaging. SCT: selective CT.

Primary imaging modality	Missed injury	ISS
SCT	Splenic laceration	16
SCT	Right 1, 3-7 rib fractures, mild pulmonary contusion, scapular fracture	8
Plain film imaging	Sub capsular liver haematoma	9

Length of Stay

The mean LOS for patients who received WBCT as an adjunct to the secondary survey was 2.4 days, compared with 1.4 days for SCT. Pairwise comparison using the Student-Newman-Keuls test showed the increased overall length of stay with WBCT compared with SCT and plain films. However, ISS was also significantly higher in this group (p<0.05).

Further subgroup analysis was performed to determine if imaging type influenced LOS independent of ISS using a one-way ANOVA. The subgroup ISS 0 (no clinically significant injuries, n=273), as the largest group, showed that WBCT was significantly associated with a longer length of stay (p<0.001). Interestingly, of the 273 patients with no clinically significant injuries (ISS 0), 156 were admitted for observation (LOS≥1), with the remainder (n=117) discharged the same day. No significant differences in LOS were detected in the two next largest groups, ISS 1 (n=28, p=0.541) and ISS 4 (n=98, p=0.197). Small numbers of patients with similar ISS precluded subgroup analysis for other ISS scores.

## Discussion

The selection of imaging modality in trauma is an important clinical issue, with WBCT having up to a threefold increase in radiation exposure compared to SCT. This decision-making process is complex, and the benefit is not always clear-cut. In certain circumstances, the combination of SCT and plain films can lead to equivalent radiation exposure [7]. Completion trauma imaging may also be required if there is a clinical change. The evidence available to clinicians to guide them in this decision-making process can be conflicting, leading to variable practice amongst clinicians, largely based on personal experience [8].

The existing evidence in this area has largely been performed in level 1 trauma centres, excluding minor trauma. A meta-analysis by Jiang et al. of imaging in major trauma patients has shown lower all-cause mortality associated with WBCT major trauma [9], with no effect on in-hospital length of stay but with reduced stays in ED. In comparison, the highly anticipated REACT-2 randomised control trial in 2016 concluded that there was no survival benefit to WBCT over SCT. This study also, however, showed no difference in time spent in the trauma room [4].

Our data confirm that the missed injury rate for clinically significant injuries is low at 1.18% (n=6) when considering a cross-section of all trauma, with each only requiring conservative treatment. This study correlates well with historical reviews showing low missed injury detection rates of between 0.3% and 2% [5,10]. Although clinically significant, none of the injuries detected on TS necessitated a significant deviation in management. Only nine patients in the SCT group and one in the plain film group had completion trauma scanning after early reassessment by admitting teams. However, three life-threatening injuries (ISS 8-16) were detected in this way.

WBCT was associated with a longer length of stay in hospital for those patients with an ISS of 0. Although likely guided by clinical concern or inadequate assessment due to drugs and alcohol, this may indicate that these patients are being observed longer than clinically necessary whilst waiting for clinically significant injuries to manifest. Another factor that may be at play is an increased number of incidental findings, which have been associated with a longer LOS in trauma [11]. More research is required to determine the cause of this increase.

This study is limited in so far as it is a retrospective, single-centre study. In addition, significant injuries that are not clinically or radiologically appreciated at the time of the tertiary survey (such as fractures that have not been fully delineated) may not be captured. Future studies would benefit from including primary care assessment and follow-up of patients to detect those injuries that may be identified in the community. The patient group assessed also necessarily excludes those patients who were bypassed or transferred to a level 1 trauma centre, introducing selection bias. Although affecting the generalisability of this study, we believe this cohort to be representative of other level 2 trauma centres and regional hospitals. This patient group, with a lower average ISS but a broad range, is currently not well represented in the existing literature.

## Conclusions

This study fills in the research gaps for patients with a broad range of injury types, as is the common presentation in level 2 trauma centres. The data indicate that early trauma assessment with a thorough primary and secondary survey and appropriate adjunct imaging leads to a low rate of injuries detected at the tertiary survey stage. No evidence could be found to show that SCT was inferior to WBCT in the rate of missed injuries in the tertiary survey. This may allow for the omission of the tertiary survey and earlier discharge in many trauma patients.

## References

[1] (2022). Major trauma in Victoria. https://www.betterhealth.vic.gov.au/health/servicesandsupport/major-trauma-in-Victoria.

[2] (2021). Patients-visitors. https://www.barwonhealth.org.au/patients-visitors.

[3] Sierink JC, Saltzherr TP, Reitsma JB, Van Delden OM, Luitse JS, Goslings JC (2012). Systematic review and meta-analysis of immediate total-body computed tomography compared with selective radiological imaging of injured patients. Br J Surg.

[4] Sierink JC, Treskes K, Edwards MJR (2016). Immediate total-body CT scanning versus conventional imaging and selective CT scanning in patients with severe trauma (REACT- 2): a randomised controlled trial. Lancet.

[5] (2021). Victorian hospitals across Gippsland, Geelong and Warrnambool hit by ransomware attack. https://www.abc.net.au/news/2019-10-01/victorian-health-services-targeted-by-ransomware-attack/11562988?nw=0&r=HtmlFragment.

[6] Stephan PJ, McCarley MC, O’Keefe GE, Minei JP (2002). 23-hour observation solely for identification of missed injuries after trauma: is it justified?. J Trauma Acute Care Surg.

[7] Wedegärtner U, Lorenzen M, Nagel HD, Weber C, Adam G (2004). [Diagnostic imaging in polytrauma: comparison of radiation exposure from whole-body MSCT and conventional radiography with organ-specific CT]. Rofo.

[8] Wutzler S, Marzi I (2016). Routine total-body CT for trauma room patients-life saver or needless radiation exposure?. J Thorac Dis.

[9] Jiang L, Ma Y, Jiang S, Ye L, Zheng Z, Xu Y, Zhang M (2014). Comparison of whole-body computed tomography vs selective radiological imaging on outcomes in major trauma patients: a meta-analysis. Scand J Trauma Resusc Emerg Med.

[10] Thomson CB, Greaves I (2008). Missed injury and the tertiary trauma survey. Injury.

[11] Andrawes P, Picon AI, Shariff MA, Azab B, von Waagner W, Demissie S, Fasanya C (2017). CT scan incidental findings in trauma patients: does it impact hospital length of stay?. Trauma Surg Acute Care Open.

